# Plant Organellar DNA Polymerases Evolved Multifunctionality through the Acquisition of Novel Amino Acid Insertions

**DOI:** 10.3390/genes11111370

**Published:** 2020-11-19

**Authors:** Antolín Peralta-Castro, Paola L. García-Medel, Noe Baruch-Torres, Carlos H. Trasviña-Arenas, Víctor Juarez-Quintero, Carlos M. Morales-Vazquez, Luis G. Brieba

**Affiliations:** 1 Laboratorio Nacional de Genomica para la Biodiversidad, Centro de Investigacion y de Estudios Avanzados del IPN, Apartado Postal 629, Irapuato CP 36821, Mexico; antolin.peralta@cinvestav.mx (A.P.-C.); libertad.garcia@cinvestav.mx (P.L.G.-M.); noe.baruch@cinvestav.mx (N.B.-T.); c.travar@gmail.com (C.H.T.-A.); victor.juarez@cinvestav.mx (V.J.-Q.); carlos.moralesva@cinvestav.mx (C.M.M.-V.); 2 Department of Chemistry, University of California, Davis, One Shields Ave, Davis, CA 95616, USA

**Keywords:** plant organellar DNA polymerases, DNA replication, DNA repair, mitochondria, chloroplast

## Abstract

The majority of DNA polymerases (DNAPs) are specialized enzymes with specific roles in DNA replication, translesion DNA synthesis (TLS), or DNA repair. The enzymatic characteristics to perform accurate DNA replication are in apparent contradiction with TLS or DNA repair abilities. For instance, replicative DNAPs incorporate nucleotides with high fidelity and processivity, whereas TLS DNAPs are low-fidelity polymerases with distributive nucleotide incorporation. Plant organelles (mitochondria and chloroplast) are replicated by family-A DNA polymerases that are both replicative and TLS DNAPs. Furthermore, plant organellar DNA polymerases from the plant model *Arabidopsis thaliana* (AtPOLIs) execute repair of double-stranded breaks by microhomology-mediated end-joining and perform Base Excision Repair (BER) using lyase and strand-displacement activities. AtPOLIs harbor three unique insertions in their polymerization domain that are associated with TLS, microhomology-mediated end-joining (MMEJ), strand-displacement, and lyase activities. We postulate that AtPOLIs are able to execute those different functions through the acquisition of these novel amino acid insertions, making them multifunctional enzymes able to participate in DNA replication and DNA repair.

## 1. Introduction

**Figure 1:**
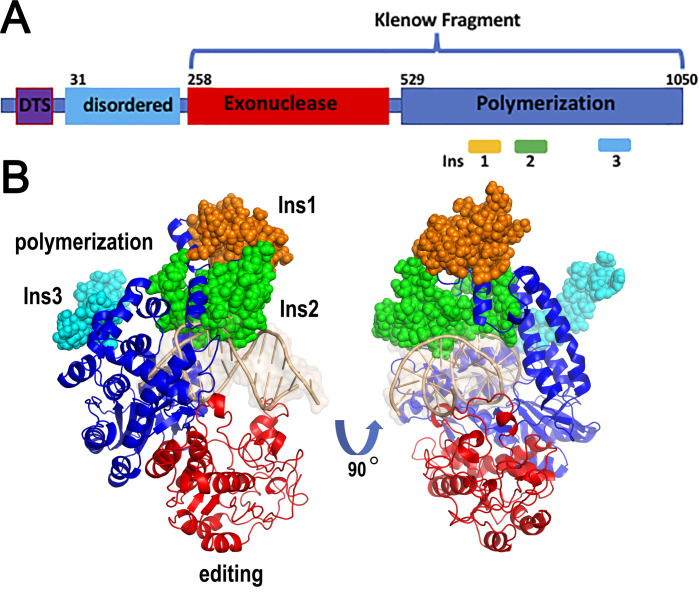
Structural architecture of AtPOLIs. (**A**) AtPOLIs, like all POPs, are modular polymerases with exonuclease (editing) and polymerization domains. AtPOLIs harbor a dual targeting sequence (DTS) for mitochondria and plastid localization and a N terminal disordered region of approximately 200 amino acids. (**B**) Homology model of AtPOLIBs: The three unique amino acid insertions in the polymerization domain of AtPOLIs are represented in a ball stick representation and colored in orange, green, and cyan.

Plant mitochondria and chloroplast are semiautonomous organelles with self-contained genomic material [1]. Their genomes encode indispensable components for phosphorylation and photosynthesis. In contrast to the circular architecture of animal mitochondrial genomes, plant mitochondrial genomes are linear molecules that present multiple isoforms due to genomic rearrangements [2,3]. Mitochondrial genomes of yeast and animals are replicated by enzymes related to T-odd bacteriophages and not by enzymes homologous to bacterial family-C DNA polymerases (DNAP) [4,5,6]. A family-A DNAP, dubbed DNAP, replicates the mitochondrial genomes of yeast and animals. DNAP is a high-fidelity enzyme able to incorporate thousands of nucleotides in a single binding event [7,8]. Thus, it was expected that plant organellar DNAP would be related to yeast and metazoans DNAP. Surprisingly, DNAPs found in plant organelles are distantly related to DNAP and are not grouped in the subfamily of yeast and animal mitochondrial DNAPs [9,10,11,12]. Because of their singularity, these DNAPs are classified into a separate group dubbed plant organellar DNA polymerases (POPs) [9,10,11,12]. Flowering plants harbor two nuclear-encoded POPs orthologues that are dually targeted to chloroplast and mitochondria [9,13,14]. In the plant model *A. thaliana*, those orthologues are named AtPOLIA and AtPOLIB [15,16]. Both polymerases share more than 70% amino acid identity [11,17] and a structural analysis illustrates that AtPOLIs have a conserved 5′–3′ polymerization and 3′–5′ exonuclease or editing domain (Figure 1A). The polymerization domain is anthropogenically associated with a cupped human right hand in which three subdomains dubbed thumb, fingers, and palm assemble the active site [18].

POPs are the sole DNAPs localized in plant organelles and in *A. thaliana*, these polymerases have evolved to function in both DNA replication and translesion DNA synthesis (TLS) [17,19]. Both AtPOLIs efficiently bypass AP sites and thymine glycol, even in the presence of an active 3′–5′exonuclease domain [17,20]. The most notorious characteristic of AtPOLIs, in comparison to bacterial DNAPs, is the presence of three insertions in their polymerization domain. Insertions 1 and 2 are located in the thumb subdomain and a homology model suggests that they are poised to interact with double-stranded DNA, whereas insertion 3 is localized at the finger´s subdomain (Figure 1) [17,20]. The abilities to execute lesion bypass in AtPOLIs reside in insertions 1 and 3. These insertions are functionally similar to insertions 2 and 3 that confer TLS capabilities to human DNAP [21,22]. However, the amino insertions of POPs and DNAP have a different structural localization and share no sequence homology [17]. Thus, plant organellar DNA polymerases harbor three unique amino acids insertions that are not present in any other DNAPs studied to date [9,10,11,12,17]. 

Although plant organellar DNA polymerases and animal mitochondrial DNA polymerases are distantly related, other components of the replication and transcription apparatus in plant and animal mitochondria like RNA polymerases, primase-helicases, and single-stranded binding proteins share sequence and structural homology with proteins from T-odd bacteriophages [6,23,24,25,26,27,28,29]. As POPs are the only DNAPs found in plant organelles, they are predicted to be essential for plant organellar DNA replication, and the impossibility to make double AtPOLI mutants indicates that at least one AtPOLI paralog is indispensable for cellular viability [15,16].

## 2. Plant Organellar DNA Polymerase as a Replicative Polymerase

Replicative eukaryotic DNA polymerases have three characteristics that define them: (1) replisome assembly, (2) accurate nucleotide incorporation, and (3) high processivity.

### 2.1. AtPOLIs Assemble a Replisome In Vitro

**Figure 2:**
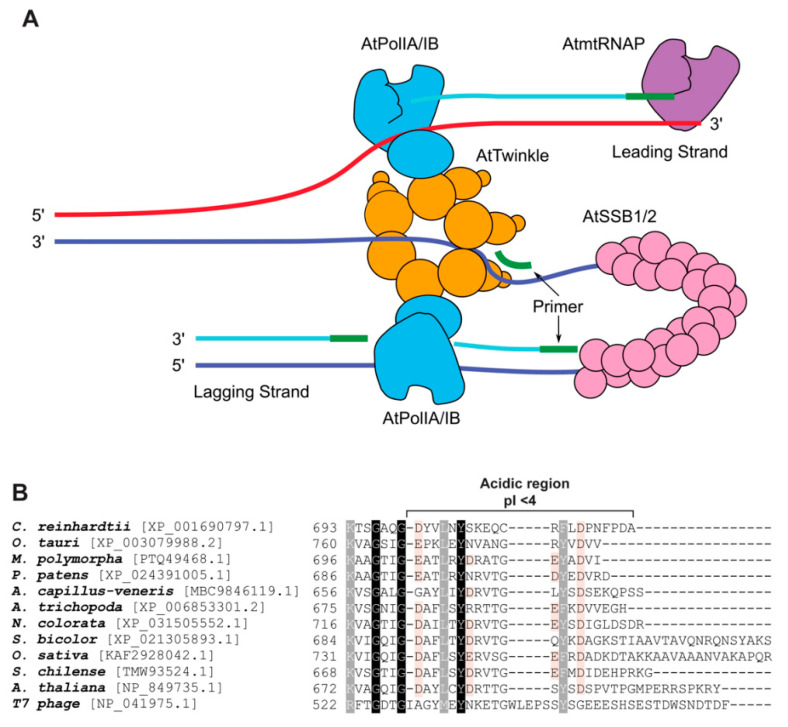
Plant mitochondria harbor proteins similar to the T7-like replisome. (**A**) Representation of the putative T7-like mitochondrial replisome of *A. thaliana*. The model predicts that in *A. thaliana,* a plant mitochondrial RNA polymerase (AtmtRNAP) may be involved in primer synthesis. AtTWINKLE is proposed to be involved in the coordination of leading and lagging DNA synthesis by specific interactions with the replicative AtPOLIs. The single-stranded segments are predicted to be covered by AtSSB1 and AtSSB2. (**B**) Amino acid sequence alignment of primases-helicases from plants in comparison to T7 primase-helicase. In both primase-helicases, the C-terminal part is highly acidic, suggesting that AtTWINKLE and AtPOLIs may exert physical interaction via charged residues as in the T7 replisome [28].

Replicative DNA polymerases execute DNA replication within a macromolecular assembly, dubbed replisome, in which helicases, primases, processivity factors, and leading and lagging DNAPs interact [30]. The minimal replisome consists of three components: a DNA polymerase, a primase-helicase, and a single-stranded binding protein, that interact during the semi-discontinuous and continuous replication of the DNA double helix [28,31]. Although the mechanisms that mediate plant organellar DNA replication are unknown, several of the proteins in *A. thaliana* that may participate in DNA replication are homologous to proteins that mediate DNA replication in bacteriophage T7 (Table 1). Seminal work by Morley and coworkers identified that in *A. thaliana*, AtPOLIs interact with the plant organellar primase-helicase (AtTWINKLE) and the organellar single-stranded binding proteins (AtSSB1 and AtSSB2), suggesting that plant mitochondrial and plastid genomes may be replicated via a T7-like replisome [23]. In the T7 replisome, the leading and lagging DNA polymerases that assemble the replication fork are in physical contact with each other via their fingers and palm subdomains [28,29]. Yeast-two-hybrid analysis confirmed that AtPOLIA interacts with itself through its exonuclease and polymerase domains, meanwhile, this interaction is not observed between AtPOLIA and AtPOLIB, and neither with AtPOLIB with itself [23]. The latter is consistent with the proposed role of AtPOLIA as the replicative DNAP in plant organelles. Another of the main interactions required to assemble a replisome is the one between the replicative helicase and the replicative polymerase. In the T7 replisome, the interaction between the leading-strand polymerase and primase-helicase is mediated by the C-terminal acidic tail (helicase domain) of the T7 primase-helicase and a region known as loading patch comprised of basic residues from the fingers subdomain of T7 DNAP. This interaction enhances the processivity of the polymerase and is essential for loading T7 DNAP into the replication fork [29,32]. T7 DNAP also contacts two adjacent subunits of the primase domain within its hexameric arrangement [28]. In *A. thaliana*, the organellar primase-helicase (AtTWINKLE) and the organellar polymerases interact in a functional manner [19,23]. AtPOLIs recognize RNA primers synthesized by AtTWINKLE that can be extended into high molecular weight products by AtPOLIs, but not by other DNA polymerases [19]. The interaction between the primase domain of AtTWINKLE and AtPOLIA is located at the fingers and exonuclease domains, like the one found in the T7 replisome. However, the interaction between the primase domain of AtTWINKLE and AtPOLIB also encompasses the polymerase domain [23]. The interaction between the helicase domain of AtTWINKLE and AtPOLIs is 2-fold weaker than that of the primase domain [23]. As mentioned before, the C-terminal tail of the T7 primase-helicase is indispensable for replisome assembly. Bioinformatic analysis of the primary structure of several TWINKLEs from plant genomes shows that a similar acidic tail is highly conserved in these enzymes. A structural model of AtPOLIs depicts a region of basic residues that is conserved among plants, suggesting that the interaction between AtPOLIs and AtTWINKLE may be similar to the interaction between T7 DNAP and T7 primase-helicase (Figure 2B). Another interaction necessary for replisome assembly is the one between the replicative polymerase and the single-stranded binding protein (SSB). The interaction between T7 SSB of the T7 DNAP is mediated through the thioredoxin binding domain (TBD) of T7 DNAP and the C-terminal tail of T7 SSB [33]. AtSSB1 interacts with AtPOLIB in vitro [23,34], although the specific residues that mediate this interaction are unknown.

### 2.2. AtPOLIs Present Moderate Fidelity

In humans, errors in mitochondrial DNA replication are associated with aging and a plethora of diseases [38]. Accordingly, replicative mitochondrial DNA polymerases from animals perform nucleotide incorporation with high fidelity. For instance, human mitochondrial DNA polymerase exhibits an average substitution error rate of ≤1.7 × 10^−6^, meaning that this polymerase makes approximately 1.7 errors per million of incorporation events [7]. This substitution rate is similar to that observed for nuclear replicative DNAPs (1 × 10^−8^ to 1 × 10^−6^) [39]. In contrast to the high fidelity exhibited by animal mitochondrial DNA polymerases, plant organellar DNA polymerases display a modest fidelity during nucleotide incorporation. AtPOLIA and AtPOLIB are 3.5- and 26-times less accurate than mitochondrial DNA polymerase [40], meaning that AtPOLIA and AtPOLIB display error rates that would generate 1 misincorporation event per 14,000 and 1800 replicated bases, respectively. The observed differences in incorporation fidelity between AtPOLIA and AtPOLIB paralogs correlates with a putative role of AtPOLIA as the replicative DNA polymerase in *A. thaliana* [15].

The moderate and low nucleotide incorporation fidelity by AtPOLIA and AtPOLIB is puzzlingly, especially because mitochondrial and plastid genomes in flowering plants present some of the slowest substitution rates in nature [41,42]. Plant genomes harbor enzymatic components like RECA or RADA, that by homologous recombination may ameliorate the misincorporation events generated by AtPOLIs [25,26,43]. However, the enzymatic mechanisms that propitiate a low substitution rate in plant organelles are unknown. Recent work suggests that MSH1, an enzyme with domains homologous to bacterial MUTS and GIY-YIG endonucleases and analogous to a MUTS/MUTL complex, is in part responsible for eliminating mutations in plant organellar genomes [44].

### 2.3. AtPOLIs Harbor an Insertion Poised to Confer Processivity

Plant mitochondrial genomes are larger than many bacterial genomes. They rank between 200 kbp to more than 5.5 Mbp [45,46], in sharp contrast with animal mitochondrial genomes that are approximately 15 kbp [47]. Although the precise nature of plant mitochondrial replication is unknown, the highly recombinant character of mitochondrial genomes and the presence of linear molecules, head-to-tail concatemers, and rosette-like structure associates with a recombination-dependent replication (RDR) mechanism [3,48,49]; whereas, the presence of a T7-like replication apparatus suggests a coordinate leader/lagging trombone-like mechanism [23]. Although both are plausible replication mechanisms, in both cases, the replicative DNAP in plant organelles is a POP. In order to avoid disruption and re-initiation of the replication fork, the replicative POP has to display high processivity. Processivity refers to the ability of DNAPs to synthesize thousands of nucleotides in a single binding event. This feature can be an intrinsic property of replicative DNAPs, as is the case for ϕ29 DNAP or yeast mitochondrial DNAPs [8,50]. However, in most cases, replicative DNAPs associate with processivity factors to increase their processivity [51]. The latter is the case of T7DNAP and HsDNAP that interact with processivity factors via specialized insertions in their thumb subdomains [27,52,53,54,55].

POPs from rice and the red algae *Cyanidioschyzon merolae* incorporate near 1000 nts per binding event [12,14]. However, this number pales in comparison to replicative DNAPs that are able to incorporate more than 10,000 nts or near 100,000 nts per binding event [50,56,57]. It is plausible that POPs, like yeast mitochondrial DNAPs, do not need accessory factors, or that they are able to exert protein–protein interactions with non-identified proteins to increase their processivity. In this sense, the unique amino acid insertions in POPs are poised to interact with processivity factors or to promote a structure able to encircle or tether along DNA (Figure 1). 

## 3. Plant Organellar DNA Polymerases Execute Translesion DNA Synthesis 

DNA lesions are byproducts of DNA replication, spontaneous errors, interaction with free radicals, and UV light. The powerhouse function of chloroplasts and mitochondria requires a steady electron flow through enzymatic complexes to produce rich-energy molecules like ATP and NADPH during photosynthesis and electron chain transport. Destabilization of electron flow generates reactive metabolic by-products known as reactive oxygen species (ROS) that react with DNA and alter its coding capabilities [58]. Live cells reduce these injuries using different repair pathways that include base excision repair (BER) and nucleotide excision repair (NER) [59,60]. However, if DNA lesions are not repaired, organisms have a DNA damage tolerance mechanism dubbed translesion DNA synthesis (TLS). In TLS, specialized DNA polymerases incorporate a nucleotide opposite the lesion and continue the replication process in order to avoid replication fork collapse [61]. Apurinic/apyrimidinic (AP) sites are the most common DNA lesions, as the result from the cleavage of modified bases by DNA glycosylases or by hydrolysis of glycosidic bonds. AP sites are strong blocks for replication and the absence of a template base makes them highly mutagenic. In nuclear genomes, DNA lesions are bypassed by specialized DNA polymerases, mostly from the family-Y, a family of DNAPs that lack 3′–5′ exonuclease or editing activity [62]. 

**Figure 3:**
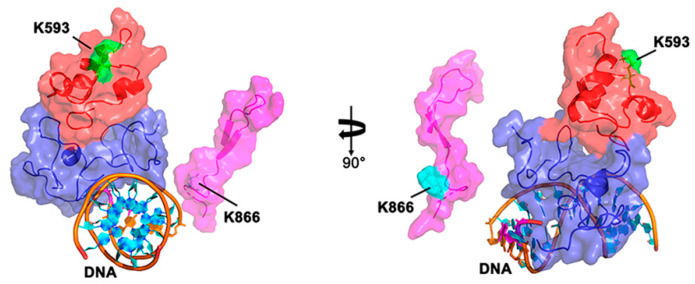
Schematic illustration of the role of insertions 1 and 3 of AtPOLIB in TLS. Homology model showing a close view of AtPOLIB in complex with a dsDNA substrate. This homology model was constructed using the molecular operating environment (MOE) software and as a template, the crystal the structure of *Bacillus* DNAP bound to dsDNA (PDB: 4DSE) [34,65]. This model shows that insertions 1 and 3 are in an optimal position to interact with dsDNA and highlights the relative positions of insert 1 (red-colored) and insert 3 (pink-colored) poised to clamp DNA. The localization of residues K593 and K866 located in insertions 1 and 3 respectively, are labeled.

POPs are the sole DNAPs in plant organelles and these polymerases have evolved TLS capabilities [17]. Both AtPOLIs efficiently bypass AP sites, even when its associated 3′–5′ exonuclease domain is active. Lesion bypass by AtPOLIs is associated with insertions 1 and 3, located in the thumb and finger subdomains, respectively (Figure 3). Since AtPOLIs are the sole DNAPs in mitochondria and chloroplasts, it is possible that the ability to bypass DNA lesions is directly linked to the acquisition of those insertions. Besides TLS at AP sites, AtPOLIs execute TLS across thymine glycol, preferentially by incorporating dAMP opposite to the lesion [20]. As is the case for an AP site, insertion 1 and insertion 3 of AtPOLIs are responsible for TLS [20]. 

Human DNAPQ (HsDNAP) is a TLS DNAP that also harbors three insertions in its polymerization domain [21,22,63,64]. However, these insertions are not related to the insertions present in POPs. HsDNAP executes TLS because of a single arginine residue (R2254) located in insertion 2 [21]. Sequence analysis of several POPs shows that insertion 1 is particularly rich in lysines [17,20]. AtPOLIB holds seven lysines in this insertion (K590, K593, K599, K605, K606, K610, and K615), whereas insertion 3 harbors two lysines (K859 and K866) [20]. From those amino acids, residues K593 and K866 are essential for TLS [20]. With basis on a homology model constructed using as a model the structure of *Bacillus* DNAP with dsDNA, the localization of residues K593 (insertion 1) and K866 (insertion 3) suggests that insertions 1 and 3 may suffer a conformational change, creating a clamp that stabilizes the primer-template during lesion bypass [20,34] (Figure 3).

## 4. Plant Organellar DNA Polymerases Are Involved in Base Excision Repair 

**Figure 4:**
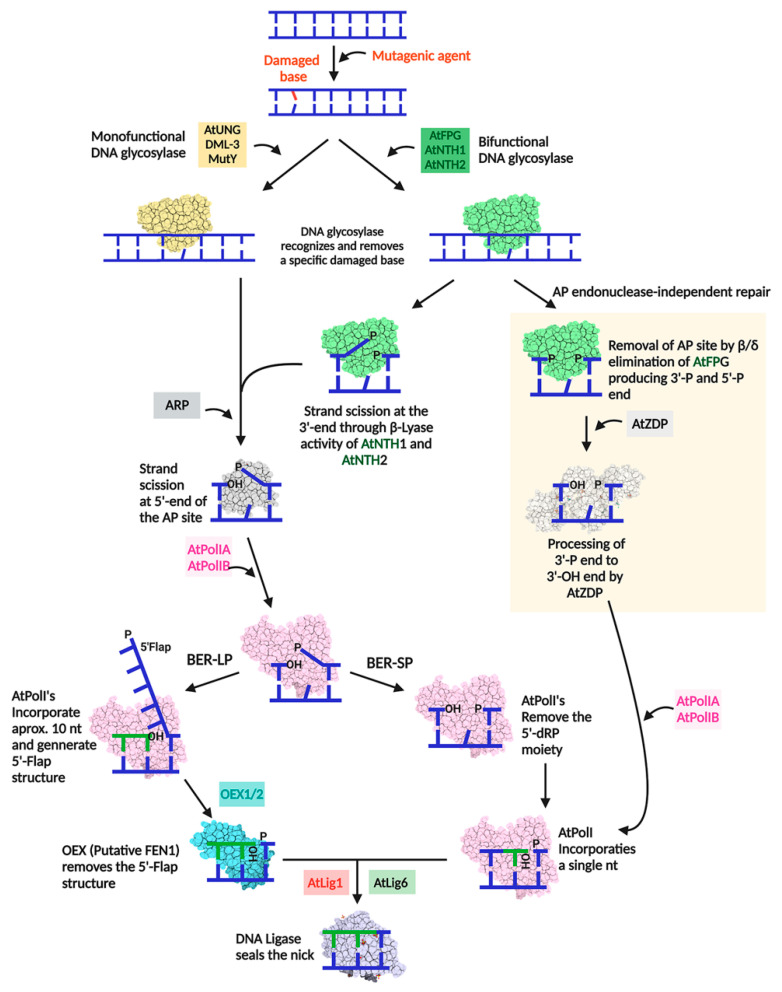
Schematic representation of the organellar BER pathway in *A. thaliana*. BER starts with a DNA glycosylase that recognizes and hydrolyzes the N-glycosidic bond of a damaged base leaving an AP site. Monofunctional glycosylases only harbor hydrolytic activity, while bifunctional glycosylases harbor both hydrolytic and lyase activities. Bifunctional DNA glycosylases, like NTH/EndoIII, cleaves the phosphodiester bond at the 3′ position of the AP site, producing a 3′-OH and a 3′-deoxyribose phosphate (3′-dRP). An AP endonuclease (ARP) processes AP sites produced by monofunctional glycosylases generating 3′-OH and 5′-dRP ends. ARP also process 3′-dRP ends produced by bifunctional glycosylases generating 3′-OH and 5′-P ends. From this point, the BER pathway is bifurcated into two sub-pathways, dubbed short (SP) and long patch (LP). During the BER-LP, the strand-displacement activity of AtPOLIs generates a 5′-flap that is cleaved by a putative FEN1 endonuclease (OEX1 and OEX2). The lyase activity of AtPOLIA or AtPOLIB is required to process the 5′-dRP moiety and incorporate a single nucleotide during BER-SP. In both sub-pathways, a DNA ligase seals the remaining nick. Alternatively, a bifunctional glycosylase, such as AtFpg, excises a DNA lesion with concomitant processing of the AP site by β, δ-elimination, creating 3′-P and 5′-P ends. The 3′-P end blocks replication and it is putatively removed by AtZDP phosphatase to leave a 3′-OH end, which is subsequently resolved by BER-SP.

Base Excision Repair (BER) is one of the most conserved DNA repair routes. This route is responsible for repairing DNA containing AP sites, 8oxoG, and uracil, among more than 30 other DNA lesions [66,67,68,69]. BER starts by a DNA glycosylase that recognizes a DNA lesion and excises its N-glycosidic bond, generating an AP site (Figure 4). This AP site can be processed by an AP endonuclease, which cleaves the phosphodiester bond at the 5′ position of the AP site, leaving a nicked DNA with 3′-OH and 5′-deoxyribosephosphate (5′-dRP) ends. At this point, BER bifurcates into two sub-pathways: short and long patch (BER-SP and BER-LP). In the short patch BER, the 5′-dRP terminus is processed to a 5′-phosphate (5′-P) by the 5′ AP lyase activity of a specialized DNA polymerase through formation of a Schiff base between a catalytic lysine and the 5′-dRP moiety, which is subsequently resolved by a *β*-elimination reaction [70]. The 5′-dRP removal makes room to accommodate a single nucleotide by the same DNA polymerase, leaving a nick between the 3′-OH end and the 5′-phosphate. Alternatively, BER-SP can be initiated by a bifunctional DNA glycosylase that harbors both glycosylase and lyase activities. This kind of DNA glycosylases (such as NTH/Endonuclease III) cleaves an AP site at the 3′ position, generating a 5′-P and a 3′-dRP site through *β*-lyase activity. The 3′-dRP can be removed by an AP endonuclease with phosphodiesterase activity [71] or it can be removed in an AP endonuclease-independent way. The latter occurs when a bifunctional DNA glycosylase (such as FPG and NEIL1) performs both base hydrolysis and AP site removal via *β*, d-elimination, generating 5′-P and 3′-P ends. Subsequently, the 3′-P is converted to a 3’-OH by a polynucleotide kinase. Finally, a DNA polymerase fills the gap with the proper nucleotides and a DNA ligase seals the nick. In most organisms, the main DNA polymerase in charge of BER-SP is DNA polymerase [72]. However, this polymerase is absent in plants [73]. On the other hand, BER-LP requires a specialized DNAP with strand-displacement activity, that incorporates several nucleotides (10–12 bases) after the processed AP site. This generates a 5′-flap structure which is resolved by a 5′-flap endonuclease or FEN1. FEN1 cleaves 2–8 nts from the 5′-dRP terminus, creating a DNA gap. The resolved 5’-flap structure generates a nick which is filled by a DNA polymerase and sealed by a DNA ligase. In plants, most of the enzymatic repertory of nuclear, mitochondria, and chloroplast BER has been studied, including BER-SP and BER-LP sub-pathways [74,75,76,77,78,79] (for a recent review, see Reference [78]). *A. thaliana* harbors all the necessary enzymes to execute BER in mitochondria and chloroplast. Moreover, the cellular localization of these enzymes in combination with functional and proteomic studies indicates that BER is a functional repair route in plant organelles [13,69,76,77,80,81,82] (Table 2) (Figure 4).

AtPOLIs harbor lyase and strand-displacement abilities that allow them to participate in BER-SP and BER-LP [84]. The catalytic amino acids necessary for lyase activity are located in insertion 1 and 3. Specifically, a lysine from insertion 1 (K593) acts as a nucleophile to form a Schiff base with a 5′-dRP moiety, propitiating its removal through a β-elimination. AtPOLIs are capable of strand-displacement that facilitates 5′-dRP removal by a flap endonuclease [84]. The acquisition of specific insertions in AtPOLIs to execute BER is reminiscent of their mammalian counterparts DNAP *β* and λ, that utilize a specific domain known as the 8 kDa lyase domain [85,86]. 

## 5. Plant Organellar DNA Polymerase Accomplish Micro-Homology-Mediated End-Joining of Double-Stranded Breaks 

DNA rearrangements in plant organelles are attributed to the repair of double-strand breaks (DSBs) [24], considered the most lethal damage to genetic information. The rate of DSBs per cell per cycle is directly related to the number of phosphorylated molecules of histone-2AX (γ-H2AX) that appear when double-strand breaks occur [87,88]. Sources that trigger DSBs are UV radiation, ROS, and replication errors [89]. To ameliorate DSBs, plant organelles have three main pathways: Homologous recombination (HR), Non-Homologous End-Joining (NHEJ), and microhomology-mediated end-joining (MMEJ) [25,26,43,90,91]. HR is abundant in plant mitochondrial genomes, especially at large repeats (>500 bp) or intermediate size repeats (between 50 to 500 bp) [92,93]. NHEJ occurs in plant organelles, although the enzymatic mechanisms that mediate this reaction are unknown [15,43,93,94]. DSBs in plant organelles can also be repaired via MMEJ using micro-homologous sequences that rank between 4 to 25 nts [15,94,95]. MMEJ, like HR, needs free 3’-OH ends tails, although they are extremely short (as short as 2 bps). MMEJ is dependent of a DNA polymerase that anneals the micro-homologous regions and uses them as a template [96,97,98]. In animals, MMEJ is limited to DNA polymerases λ, *β*, and θ [34,64,99]. DNAP θ plays an essential role in MMEJ, especially if homologous recombination is defective [100,101,102]. This enzyme is a family-A DNAP with an associated N-terminus helicase domain and a central region with an unknown function [103,104]. Both helicase and polymerase domains are required for MMEJ [105,106,107,108]. The polymerization domain of DNAP θ contains three amino acid insertions. Insertion 2 is indispensable for synapsis formation and annealing steps via residue Arg2254 [21,64]. 

In plant organelles, POPs execute MMEJ in a similar fashion to DNAP θ [34]. Like DNAP θ, POPs harbor three insertions that are not related to the insertions present in DNAP θ. Biochemical data showed that residues K590A and K593A from insertion 1, and K866A from insertion 3, are necessary for MMEJ [34]. Moreover, POPs can execute MMEJ in synthetic substrates containing one, two, or three mismatches [34]. Synapsis formation during MMEJ is a crucial step to permit the correct annealing of microhomologies [109] and it is proposed that POPs assemble a dimer in a *trans*, permitting the DNAP monomers to grasp partial single-stranded DNA (pssDNA) to join them and to form a double-stranded DNA [34,64,105]. 

MMEJ in plant organelles is regulated by ssDNA binding proteins: whirlies (WHY1, WHY2, and WHY3) [110], organellar single-stranded binding proteins (OSB1, OSB2, OSB3, and OSB4) [111], and the canonical single-stranded DNA binding proteins (mtSSB1 and mtSSB2) [34,37,94,110]. 

**Figure 5:**
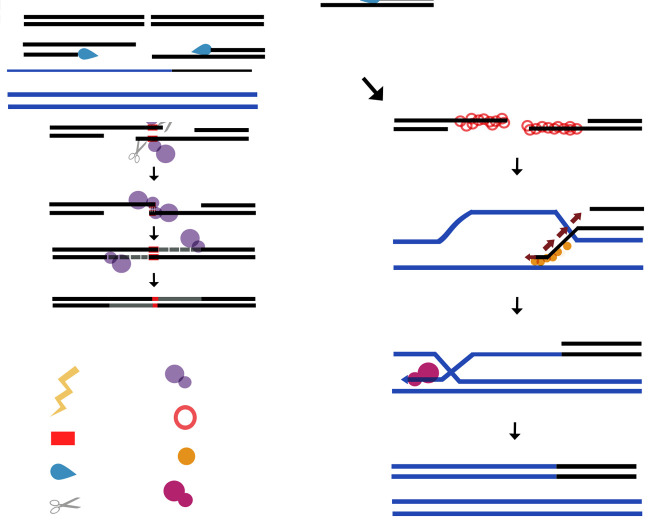
Plant mitochondria employ different mechanisms to repair DSBs. (**A**) DNA repair by HR. Repair of double-strand breaks is initiated by resection enzymes that generate a 3′-OH end. The length of the 3′OH end can be long or short. In plant mitochondria, a long 3′-OH (right panel) is protected by single-stranded binding proteins like SSBs, WHY, or OSBs. AtRECAs, with the help of accessory proteins, displace the single-stranded binding proteins and initiate the search for homologous sequences. After the strand invasion, DNA synthesis is initiated by an organellar DNA polymerase. Finally, a ligation step is necessary to seal the break. (**B**) DNA repair by MMEJ. In short 3′-OH (left panel) that contain micro-homologous sequences, DNA polymerase anneals two strands by *trans* complementation. This mechanism is known as MMEJ. These short sequences require a minimum of two base pairs to bind and extend them by the same DNA polymerase. In the organelles of *A. thaliana,* MMEJ is executed by AtPOLIA and AtPOLIB.

In knock-out mutants *why2* or *osb1*, there is an increase in the number of micro-homologous regions [110], indicating a role of those proteins in the maintenance and protection of organellar DNA. There is no physical interaction between WHYs and OSBs with POPs, however, WHYs and OSBs bind ssDNA at a nanomolar range [34,110,111]. The strong binding of WHY and OSBs to ssDNA establishes a competition between POPs and WHY/OSB proteins for the 3′-OH tail generated during the resection step. If WHY or OSB captures the ssDNA tail, the ssDNA will be protected from exonucleolytic processes until the HR machinery is recruited [110]. On the other hand, if POPs occupy the 3’-OH tail, they will be able to execute partially single-stranded DNA (pssDNA) alignments using micro-homologous sequences (MMEJ) (Figure 5) [34]. 

## 6. Conclusions

POPs are classified in a novel class of DNAPs exclusively present in protozoan and plants. Protozoan, algae, and non-vascular plants harbor a single gene encoding for a POP, whereas POPs in flowering plants, with the exception of members of the *Solanaceae* family, are a duplicated gene [9,112]. The plant model *A. thaliana* contains two POPs: AtPOLIA and AtPOLIB. Sharing 70% of amino acid identity, these two DNA polymerases appear to have different roles during organellar DNA replication. In vivo evidence shows that knocking out either of these polymerases results in a non-lethal phenotype, suggesting that the bulk of DNA replication can be carried out by any AtPOLIs, however at least one AtPOLIs is necessary for cell viability [15,16]. Both AtPOLIs exhibit less fidelity for nucleotide incorporation than human mitochondrial Pol γ. However, the error rate of AtPOLIA is 7-fold lower than that of AtPOLIB [40]. DNA polymerases are modular proteins, for instance, their DNA polymerization domain has suffered the integration of modules with 3′–5′ exonuclease, helicase, or histidinol phosphatase domains, and this modular nature has allowed the incorporation of diverse functions [113]. AtPOLIs execute multiple functions by virtue of three amino acids insertions. Insertions 1 and 3 are responsible for: 5′-dRP lyase [84], micro-homology-mediated end-joining (MMEJ) [34], and TLS [17], whereas insertion 2 may play a role in conferring processivity. Thus, AtPOLS POPs are an example of biological innovation through the acquisition of novel insertions. We postulate that being the sole DNAPs in plant mitochondria and chloroplasts, AtPOLIs have evolved as multifunctional DNAPs to deal with the various damages and distortions found in DNA, and that, in the nucleus, these activities are carried out by a machinery composed of several DNAPs and accessory proteins, the latter at the expense of exhibiting a moderate fidelity for nucleotide incorporation. 

## Figures and Tables

**Table 1:** Enzymatic components of a minimal organellar replisome.

Enzyme	Gene	Activity	TAIR ID	Localization ^+^	References for Cellular Localization
Family A DNA polymerase	*AtPOLIA*	DNA polymerase	AT1G50840	M, C	[13,35]
Family A DNA polymerase	*AtPOLIB*	DNA polymerase	AT3G20540	M, C	[13,35]
DNA primase-helicase	*AtTWINKLE*	DNA primase DNA helicase	AT1G30680	M, C	[35,36]
SSB1	*AtSSB1*	Single-stranded binding protein	AT4G11060	M, C	[35]
SSB2	*AtSSB2*	Single-stranded binding protein	AT3G18580	M	[35,37]

^+^ M: Mitochondrion, C: Chloroplast.

**Table 2:** Base Excision Repair proteins present in mitochondria and chloroplasts in *A. thaliana*.

Enzyme	Gene	TAIR ID	Mitochondrial (Predicted TargetP [83])	Chloroplast (Predicted TargetP [83])	Reference for	Substrate
Uracil DNA glycosylase	*AtUNG*	AT3G18630	+ (Experimental)	+ (Predicted)	[76]	U, 5-FU
Endonuclease III 1	*AtNTH1*	AT2G31450	+ (Predicted)	+ (Experimental)	[77]	Tg, 5-hC, 5-hU, Fapy lesions
Endonuclease III 2	*AtNTH2*	AT1G05900	+ (Predicted)	+ (Experimental)	[77]	Tg, 5-hC, 5-hU, Fapy lesions
Formamidopyrimidine DNA Glycosylase	*AtFPG*	AT1G52500	+ (Predicted)	- (Predicted)	n.d	Fapy lesions and 8oxoG
5-meC and thymine-DNA glycosylase	*DML-3*	AT4G34060	- (Predicted)	- (Predicted)	n.d	T:G, U:G, 5-MeC, halogenated pyrimidines, 5-FU, Tg:G
Adenine DNA Glycosylase	*MutY*	AT4G12740	+ (Predicted)	+ (Predicted)	n.d	A across 8oxoG
Polynucleotide 5′-Kinase/3′-Phosphatase	*AtZDP*	AT3G14890	+ (Predicted)	+ (Predicted)	n.d	Dephosphorilates 3′-P ends producing 3′-OH
Xth Endonuclease	*ARP*	AT2G41460	+ (Predicted)	+ (Experimental)	[77]	AP sites, DHU, α-dA
Family A DNA polymerase	*AtPOLIA*	AT1G50840	+ (Experimental)	+ (Experimental)	[13]	Lyase and strand-displacement
Family A DNA polymerase	*AtPOLIB*	AT3G20540	+ (Experimental)	+ (Experimental)	[13]	Lyase and strand-displacement
FEN1	*OEX1*	AT3G52050	+ (Predicted)	- (Predicted)	n.d	5′-Flap structure
FEN1	*OEX2*	AT1G34380	- (Predicted)	+ (Predicted)	n.d	5′-Flap structure
ATP-dependent DNA Ligase	*AtLig1*	AT1G08130	+ (Experimental)	- (Experimental)	[82]	Nick-containing DNA
ATP-dependent DNA Ligase	*AtLig6*	AT1G49250	- (Predicted)	+ (Predicted)	n.d	Nick-containing DNA
